# Gambling in Malaysia: an overview

**DOI:** 10.1192/bji.2020.55

**Published:** 2021-05

**Authors:** Balan Rathakrishnan, Sanju George

**Affiliations:** 1 Associate Professor, Faculty of Psychology and Education, Malaysia University of Sabah (UMS), Kota Kinabalu, Malaysia. Email: rbhalan@ums.edu.my; 2 Professor of Psychiatry and Psychology, Rajagiri School of Behavioural Sciences and Research, Rajagiri College of Social Sciences (Autonomous), Kochi, Kerala, India. Email: sanjugeorge531@gmail.com

**Keywords:** Gambling, law, Malaysia, research

## Abstract

Many forms of gambling are legal and popular in Malaysia. Despite this, in Malaysia, research into gambling is limited and there is no coherent strategy to tackle gambling-related harms. This paper summarises the gambling landscape of Malaysia, law governing gambling and research done so far and gives some recommendations on the way forward.

Malaysia is a South East Asian country with a population of approximately 32.37 million people. Malaysia gained independence from the British Empire in 1963. Islam is the predominant religion (61.3%), followed by Buddhism (19.8%), Christianity (9.2%) and Hinduism (6.3%), and the rest practice traditional Chinese religions.^1^ Gambling is forbidden under Islamic law (Sharia law) so most Muslims do not engage in legal gambling. As Malaysia has a multi-ethnic population, with Malay making up 63%, Chinese making up approximately 25% and those with Indian ancestry making up 12% of the total 32.37 million population, it is these latter groups who gamble more through legal means and who spend more on gambling.^2,3^

‘Gambling disorder’ sits alongside the more traditional substance addictions in DSM-5. In ICD-11, the ICD-10 term ‘pathological gambling’ is replaced by ‘gambling disorder’.

## Gambling in Malaysia

It would appear that gambling was ‘brought’ to Malaysia by Chinese merchants in the 19th century. Gambling, both legal and illegal forms, is very popular in Malaysia.^4^ Some forms of gambling, such as lotteries, casino games and horse racing, are legal in Malaysia, whereas all forms of sports betting (at bookmakers) and online gambling are illegal. Gambling is legal only if a license or permit has been granted by the authorities – the Unit Kawalan Perjudian (Betting Control Unit) of the Ministry of Finance. Lotteries in Malaysia are allowed under the Lotteries Act 1952. Currently, there are six legal lotteries (all privately owned) in Malaysia). Alongside these, there exist several illegal lottery businesses and it was estimated that in 2018 ‘Malaysia's illegal lottery business generated about 60 percent more revenue than the six legal operators combined’.^5^

There is only one legal land-based casino in Malaysia. This privately owned casino was set up in the 1970s in a very ‘Las Vegas style’ and is open 24 h a day but denies entry to Muslims and those under 21 years of age. This casino offers over 400 types of electronic table games, 3000 slot machines and 30 tables with games such as blackjack, tai sai, roulette and boule.

Horse racing was introduced in Malaysia by the British during the 1800s and currently there are three racecourses and betting on horses is legal. All three racecourses are privately owned and are regulated by the Racing Act 1961.^6^

Despite being illegal, online gambling has been increasing in popularity over the past few years in Malaysia. Betting on badminton and football (mostly English football – the Premier League) is immensely popular. Technological advancements have made online gambling opportunities more accessible and more affordable. Although illegal, international betting sites accept customers from Malaysia and process deposits and withdrawals in ringgits (RM, the Malaysian currency).

## Gambling laws in Malaysia

There are three major legal frameworks that dictate gambling laws in Malaysia: the Betting Act 1953, the Common Gaming Houses Act 1953 and Shariah law. The Betting Act 1953 (with several further amendments) is the most important of these.^7^ This Act bans all forms of gambling unless the company has a legal license to operate and covers telecommunications and other means of transmitting bets between customers and betting houses. As per this Act, anyone caught running a betting house or caught in one will be penalised with a RM200 000 fine and 5 years in jail.

The Common Gaming Houses Act 1953 (with further amendments) is more inclusive than the Betting Act in its coverage of types of gambling.^7^ The Common Gaming Houses Act defines gaming as: ‘the playing of any game of chance or of mixed chance and skill for money or money's worth’. In the 2020 budget plan for Malaysia,^8^ the Finance Minister announced an increase in punishments for both illegal gamblers and gambling operators. The maximum penalty for those who gamble illegally was increased 20-fold, from RM5000 to RM100 000, and a minimum jail sentence of 6 months was introduced.

Islam being the predominant religion, Malaysia also recognises Sharia law and Sharia (or Syariah) courts. Non-Malays (mostly ethnic Chinese and Indian) are not bound by Sharia law but by the secular legal system. All forms of gambling are forbidden under the Sharia law.

## Gambling research in Malaysia

Gambling research in Malaysia has been limited.

Tan et al^3^ analysed data from 6117 non-Muslim households for sociodemographic determinants of gambling participation and gambling expenditure. They found that the sociodemographic factors associated with higher levels of gambling expenditure were being young, Chinese, having lower education levels and higher income, and being from paternal-headed families.

Two hundred patrons of the only casino in Malaysia were examined for their demographics, gambling behaviour and factors contributing to gambling decisions.^9^ It was found that marketing activities such as promotions, services, positioning and winnings predicted greater gambling behaviour, whereas psychological variables such as motivation, personality, perception and cognition did not.

Loo & Ang^10^ studied the prevalence of problem gambling in Malaysia's largest state (Selangor, with a population of 5.6 million) and found that 4.4% of the general population were problem gamblers and 10.2% were moderate-risk gamblers.

Loft & Loo^11^ analysed sleep difficulty, sleep habits, arousability, self-regulatory capacity and problem-gambling severity in 59 treatment-seeking gamblers and noted that self-regulatory capacity mediated the relationship between problem gambling and sleep difficulty, and that it was a significant mediator between problem gambling and negative sleep habits.

In a more recent study, Sheela et al^12^ looked at 2265 Malaysian adolescents and found that around 30% of them participated in some form of gambling over a 12-month period. They also noted that parental gambling, being male and high-risk behaviours were associated closely with adolescent gambling.

## Treatment services for gamblers in Malaysia

There are no structured gambling treatment facilities in the public sector in Malaysia. Several private rehabilitation centres offer residential and out-patient help for problem gamblers. There are no offline Gamblers Anonymous (GA) meetings in Malaysia but online access to GA is possible. We are aware that private psychiatric hospitals and addiction specialists offer individual psychotherapies (mostly cognitive–behavioural therapy) for problem gamblers and supportive psychotherapy to their families.

Anecdotes suggest that most people with gambling-related problems go unrecognised and untreated. Problems often only come to light when they are severe or when the gambler comes to the attention of the legal system for gambling-related debt, bankruptcy, financial fraud, domestic violence and other crimes.

## The way forward

The issue of gambling in an Islamic country like Malaysia is fraught with difficulties, unlike in more secular countries. This is because of Malaysia's unique dual system of law – the Sharia-governed Syariah courts for the nation's Muslims (over half of the total population), which strongly oppose gambling, and secular law that is less rigid in its take on gambling.

First, there needs to be a wider debate on whether gambling ought to be banned altogether in Malaysia or whether it can be liberalised (at least in certain forms) and tightly regulated. Such a debate needs to involve multiple stake holders, including policy makers, academics, healthcare professionals and the gambling industry. At the very least, existing legislation needs to be updated to include online gambling in its various guises. Second, more needs to be done to minimise gambling-related harm and it might be best to start with public health approaches such as awareness-raising campaigns about various aspects of gambling, its potential for harm, signs and symptoms, how to seek help, banning and enforcement thereof of gambling advertisements and promotions (in both print and online media) and the increasing in-counter-advertising whereby this type of advertisement focuses on certain issues, persons or products to take a stand against other adverstisements in regards to controversial topics. Third, treatment services for Malaysia's problem gamblers and their families need to be expanded into the public sector as well. Given that gambling is illegal and banned under Sharia law in Malaysia, it is best viewed as a mental health problem with adverse public health impact. Healthcare service providers and health policy makers need to work together to provide psychological and psychiatric treatments in the community and hospital settings. This should include rehabilitation centres for those addicted. Fourth, more research needs to be done into the prevalence of problem gambling and gambling-related harms. Finally, we believe that developing a national gambling strategy and having an independent body to oversee it will aid the translation of the above suggestions into reality.
